# Complement Opsonization Promotes Herpes Simplex Virus 2 Infection of Human Dendritic Cells

**DOI:** 10.1128/JVI.00224-16

**Published:** 2016-04-29

**Authors:** Elisa Crisci, Rada Ellegård, Sofia Nyström, Elin Rondahl, Lena Serrander, Tomas Bergström, Christopher Sjöwall, Kristina Eriksson, Marie Larsson

**Affiliations:** a Division of Molecular Virology, Department of Clinical and Experimental Medicine, Linköping University, Linköping, Sweden; b Division of Clinical Microbiology, Linköping University Hospital, Linköping, Sweden; c Division of Infectious Diseases, Department of Clinical and Experimental Medicine, Linköping University, Linköping, Sweden; d Department of Infectious Disease, Institute of Biomedicine, University of Gothenburg, Gothenburg, Sweden; e AIR Rheumatology, Department of Clinical and Experimental Medicine, Linköping University, Linköping, Sweden; f Department of Rheumatology and Inflammation Research, University of Gothenburg, Gothenburg, Sweden

## Abstract

Herpes simplex virus 2 (HSV-2) is one of the most common sexually transmitted infections globally, with a very high prevalence in many countries. During HSV-2 infection, viral particles become coated with complement proteins and antibodies, both present in genital fluids, which could influence the activation of immune responses. In genital mucosa, the primary target cells for HSV-2 infection are epithelial cells, but resident immune cells, such as dendritic cells (DCs), are also infected. DCs are the activators of the ensuing immune responses directed against HSV-2, and the aim of this study was to examine the effects opsonization of HSV-2, either with complement alone or with complement and antibodies, had on the infection of immature DCs and their ability to mount inflammatory and antiviral responses. Complement opsonization of HSV-2 enhanced both the direct infection of immature DCs and their production of new infectious viral particles. The enhanced infection required activation of the complement cascade and functional complement receptor 3. Furthermore, HSV-2 infection of DCs required endocytosis of viral particles and their delivery into an acid endosomal compartment. The presence of complement in combination with HSV-1- or HSV-2-specific antibodies more or less abolished HSV-2 infection of DCs. Our results clearly demonstrate the importance of studying HSV-2 infection under conditions that ensue *in vivo*, i.e., conditions under which the virions are covered in complement fragments and complement fragments and antibodies, as these shape the infection and the subsequent immune response and need to be further elucidated.

**IMPORTANCE** During HSV-2 infection, viral particles should become coated with complement proteins and antibodies, both present in genital fluids, which could influence the activation of the immune responses. The dendritic cells are activators of the immune responses directed against HSV-2, and the aim of this study was to examine the effects of complement alone or complement and antibodies on HSV-2 infection of dendritic cells and their ability to mount inflammatory and antiviral responses. Our results demonstrate that the presence of antibodies and complement in the genital environment can influence HSV-2 infection under *in vitro* conditions that reflect the *in vivo* situation. We believe that our findings are highly relevant for the understanding of HSV-2 pathogenesis.

## INTRODUCTION

Worldwide, herpes simplex virus 2 (HSV-2) is one of the most common sexually transmitted infections, with a high seroprevalence, over 50% in developing countries (1, 2). Many infected individuals are asymptomatic, and shedding of HSV-2 in the genital tract can occur without any clinical symptoms (3). Notably, several studies indicate that preexisting genital herpes enhances the acquisition, transmission, and progression of human immunodeficiency virus type 1 (HIV-1) (1–4). The innate immune response of the genital tract is the first line of defense against sexually transmitted viruses, such as HSV-2 (4). In the genital mucosa, epithelial cells are primary targets of HSV-2 infection (1), but mucosal immune cells, such as dendritic cells (DCs), can also become infected by HSV-2 (5). The envelope of HSV-2 contains an array of viral glycoproteins that are involved in infection or immune evasion (6, 7). HSV-2 glycoprotein C (gC) binds complement 3b (C3b) (7–11), which provides protection against complement-mediated virus neutralization, i.e., destruction (9, 12). HSV-2 gC facilitates virus entry by attaching the viral particle to host cell surface heparin sulfate and heparin (13), and the absence of gC sensitizes HSV-2 to lysis through the classical complement pathway in epithelial cells (14).

It is clear from *in vivo* studies in different mouse models that the complement pathway plays an important role in HSV infection (15–17). Complement proteins are present in vaginal secretions (2) and seminal plasma (SP) (18, 19) and during an HSV-2 infection, the viral particles should be complement coated (9, 10), which might influence the infection and activation of the immune responses. Besides complement, the genital secretions contain antibodies (Abs) that influence the mucosal immune response (20, 21). It is possible that preexisting HSV-1 antibodies play a role in protecting individuals from acquiring HSV-2 or in the clinical manifestations of HSV-2 infection (22–24). Individuals with HSV-1 immunity tend to remain asymptomatic for HSV-2 disease and to have their first clinical manifestation of genital herpes only after experiencing an immunosuppressive event (25).

Only a few studies on the interaction between HSV-2 and human DCs exist, and they were performed using monocyte-derived DCs (MDDCs) (5, 26, 27). HSV-2 induces a productive viral infection in MDDCs (5) and apoptosis in both infected and bystander cells (26). In DCs, infectious HSV-2 triggers the release of proinflammatory cytokines, most notably tumor necrosis factor alpha (TNF-α), but also interleukin 6 (IL-6) (26, 27) and antiviral factors, such as beta interferon (IFN-β) (27). Other effects exerted by HSV-2 on MDDCs include increased expression of aldehyde dehydrogenase member A1 (27) and impaired antigen presentation (26).

The aim of this study was to examine the effects of opsonization of HSV-2, i.e., with complement alone or with complement and HSV-specific antibodies, exerted on the viral infection of immature monocyte-derived DCs and the cells' ability to mount inflammatory and antiviral responses to the viral exposure. HSV-2 that was complement opsonized, both by human serum (HS) and by seminal plasma, produced enhanced infection of DCs and greater productive infection than free, nonopsonized HSV-2. Furthermore, opsonization gave rise to significantly higher gene expression of all inflammatory and antiviral factors tested, but at the protein level, these differences between free and complement-opsonized HSV-2 were not as clear as at the gene level. The presence of complement and HSV-1- or HSV-2-specific antibodies decreased infection, inflammation, and antiviral responses in DCs. HSV-2 infection of DCs required endocytosis and endosomal acidification, as inhibition of these cellular events decreased infection. The enhanced infection induced by complement-opsonized virions required functional complement receptor 3 (CR3). This work clearly demonstrates the importance of studying HSV-2 infection under conditions that reflect the *in vivo* situation, i.e., virions covered in complement fragments or complement fragments and antibodies.

## MATERIALS AND METHODS

### Reagents.

The DC culture medium, RPMI 1640 (GIBCO, Sweden), was supplemented with 2 mM glutamine, 20 μg/ml gentamicin (Gibco), 10 mM HEPES (Gibco), and 1% human plasma. Recombinant human granulocyte-macrophage colony-stimulating factor (GM-CSF) (100 U/ml; Genzyme) and recombinant human IL-4 (300 U/ml; R&D Systems, Minneapolis, MN, USA) were utilized for *in vitro* propagation of DCs. Vero cells (ATCC, United Kingdom) were cultured in Dulbecco's modified Eagle's medium (DMEM) (Gibco) with 10% fetal calf serum (FCS), 2 mM glutamine, 20 μg/ml gentamicin, and 10 mM HEPES.

### Propagation of monocyte-derived dendritic cells.

Whole blood was obtained from volunteers, as well as from four individuals with a diagnosis of systemic lupus erythematosus (SLE), based on the recent classification criteria (28), with an rs1143679 (R77H) mutation in CD11b (29) as described previously (30). Approval was granted by the Linköping University ethical committee for human research (ethical permits M173-07 and M75-08/2008), and the research was done in accordance with EU guidelines and the Declaration of Helsinki. Peripheral blood mononuclear cells (PBMC) were separated by density gradient centrifugation on Ficoll-Hypaque (Amersham Pharmacia Biotech, Piscataway, NJ, USA) and incubated on tissue culture dishes (BD) for 1 h at 37°C to allow adherence of the DC progenitors before the nonadherent cells were removed by washing with RPMI. The progenitors were differentiated into immature monocyte-derived DCs (referred to below as immature DCs) by adding 100 U/ml GM-CSF and 300 U/ml IL-4 at days 0, 2, and 4 of culture. On day 5, the DCs were assessed for expression of CD14 and CD83 markers as quality control and then used for experiments.

### Virus propagation and titration.

HSV-2 stock was prepared in African green monkey kidney (GMK) cells cultured in Eagle's minimal essential medium (MEM) supplemented with 10% FCS as previously described (31). The HSV-2 strain used was strain 333. UV inactivation of HSV-2 was accomplished by exposing the viruses to UV light for 0.5 h.

### Opsonization of HSV-2.

Complement opsonization of HSV-2 was done by incubation of virions with an equal volume of HS or SP (ethical permits M173-07 and M75-08/2008). Different types of HS were used for virus opsonization: HSV-1- and HSV-2-seronegative-serum-opsonized virus (C-HSV-2), HSV-1-seropositive-serum-opsonized virus (C1-HSV-2), or HSV-2-seropositive-serum-opsonized virus (C2-HSV-2). The HS was tested for HSV antibodies using HerpeSelect 1 enzyme-linked immunosorbent assay (ELISA) IgG and HerpeSelect 2 ELISA IgG kits (Focus Diagnostics, Cypress, CA, USA). We utilized nine HSV-seronegative sera, eight HSV-2^+^ sera, and eight HSV-1^+^ sera for the experiments. For experiments with seminal plasma, we used samples from four HSV-1- and HSV-2-seronegative donors. Free virus (F-HSV-2) was treated with medium alone, and mock treatment (medium alone) was used as a negative control. Additionally, DCs treated with serum or seminal plasma were used as a negative control. All groups were incubated for 1 h at 37°C and then directly used in the HSV-2 infection experiments. Heat inactivation of the complement (HI-C) was done by incubation of human serum or seminal plasma at 56°C for 1 h.

### HSV-2 infection of dendritic cells.

Immature DCs (0.5 × 10^6^) were mock infected or infected with F-HSV-2, C-HSV-2, C1-HSV-2, or C2-HSV-2 at a multiplicity of infection (MOI) of 1 to 3 for 2 h at 37°C in RPMI alone or in 1% plasma from HSV-seronegative donors. The different groups of DCs were then washed and cultured in 1% plasma from HSV-seronegative donors for a total of 6 h or 24 h. The DCs were harvested, washed, and lysed with Bioline RLY lysis buffer (Bioline, United Kingdom) for RNA extraction or fixed with 4% paraformaldehyde (PFA) for 10 min at 4°C for flow cytometry staining.

### Viral binding and endocytosis.

To evaluate the binding and uptake of virus particles, DCs were incubated for 2 h at 37°C and at 4°C. Cells were collected, counted, and resuspended in distilled water and stored at −20°C. To assess the number of DNA copies, viral DNA was extracted with a Qiagen EZ1 Virus Mini 2.0 extraction kit. A specific PCR was performed using a Quantifast ROX vial kit (Qiagen, Sweden) or Takyon No ROX Probe Master Mix dTTP (Eurogentec S.A., Belgium) with HSV-2 gG forward primer (AGA TAT CCT CTT TAT CAT CAG CAC CA) and HSV-2 gG reverse primer (TTG TGC CAA GGC GA) and a probe (CAG ACA AAC GAA CGC CG) (32).

To determine whether the HSV-2 infection of DCs was dependent on acidification, we used the acidification inhibitors NH_4_Cl (40 mM; Sigma) and bafilomycin A1 (BAF) (50 nM; Sigma). Additionally, the requirement for endocytosis was assessed using cytochalasin D (CCD) (10 μM; Sigma); clathrin-mediated endocytosis was assessed using chlorpromazine (CP) (6.25 μg/ml; blocks clathrin-mediated entry; Sigma), and protein transport was assessed with monensin (Mon) (4 μl/ml; BD). All the agents were used 30 min before infection of the DCs.

### Assessment of productive HSV-2 infection of DCs.

Supernatants from the HSV-2 infection experiments were harvested at 6 h and 24 h, and the viral yields were quantified using a modified plaque assay method (33) on Vero cells. Briefly, DC supernatants were incubated in 2- or 10-fold dilutions in 24-well plates with a confluent monolayer of Vero cells for 1 h at 37°C. After washing, the plates were coated with complete medium mixed with 2% agarose (1:1) and incubated for an additional 3 or 4 days before assessing PFU. Mock treatment and UV-inactivated HSV-2 (UV-HSV-2) were used as negative controls. All samples were tested in duplicate or triplicate.

### Total-RNA extraction, reverse transcription, and quantitative PCR (qPCR).

Total RNA from DCs exposed to mock treatment, F-HSV-2, C-HSV-2, C1-HSV-2, and C2-HSV-2 was extracted using an Isolate II RNA minikit (Bioline, United Kingdom), and total cDNA was produced with a SuperScript III Reverse Transcriptase First Strand cDNA Synthesis kit (Invitrogen, Carlsbad, CA, USA). Quantification of gene transcripts was performed with the SensiFast SYBR Hi-Rox kit (Bioline, United Kingdom) using a 7900HT Fast Real Time PCR system with 7900 System SDS 2.3 software (Applied Biosystems, Sweden). β-actin and glyceraldehyde-3-phosphate dehydrogenase (GAPDH) were used as housekeeping genes for reference, as described by Vandesompele et al. (34). The primers were purchased from CyberGene AB, Stockholm, Sweden (for the primer sequences, see Table S1 in the supplemental material). To compensate for variation between plates, values were normalized as described by Rieu and Powers (35).

### Assessment of inflammatory factors by ELISA.

Levels of TNF-α, IL-6, IFN-α (Mabtech, Sweden), and IFN-β (VeriKine kit; PBL Assay Science, USA) proteins were assessed in supernatants from DCs that were mock infected or infected with F-HSV-2, C-HSV-2, C1-HSV-2, or C2-HSV-2 for 24 h. In addition, thesfactors were examined in supernatants from SP-opsonized-HSV-2 (SP-HSV-2)-infected DCs at 24 h and for endocytosis studies at 6 h. All the ELISAs were performed following the manufacturer's instructions.

### Flow cytometry.

The quality of immature DCs was assessed by staining with anti-human CD83 and CD14 phycoerythrin (PE)-conjugated antibodies (BD). DCs were used if their purity was more than 95% and their expression of CD14 and CD83 was less than 10%. To evaluate the level of HSV-2 infection in immature DCs, cells were permeabilized with phosphate-buffered saline (PBS) containing 0.2% saponin and 0.2% FCS and incubated with polyclonal Ab (PAb) against HSV-2 (identifying all major glycoproteins in the viral envelope and at least one core protein; B0116; Dako, Denmark) or monoclonal Ab (MAb) against HSV-2-infected cell protein 8 (ICP8) (4E6) (Santa Cruz Biotechnology, USA), followed by fluorescein isothiocyanate (FITC)- or Alexa 488-conjugated secondary Ab (Abcam). A Zombie Aqua Fixable Viability kit (BioLegend) was used to discern viable/dead cells. Additionally, PE-conjugated MAb against CD11b/Mac-1 (BD) was used to evaluate the presence of CD11b receptor on DCs. The samples were assessed by flow cytometry (FACSCanto; BD) and analyzed with FlowJo (Treestar, Ashland, OR, USA).

### CD11b protein knockdown in dendritic cells by siRNA.

Knockdown of CD11b protein with small interfering RNA (siRNA) in immature DCs was done by transfecting immature DCs at day 2 or 3 of culture with siRNA using the transfection reagent DF4 (Dharmacon) or HiPerFect (Qiagen), respectively. The transfection reagents were removed, and the cells were used for experiments 2 days after transfection. The siRNA (Smart pool; Dharmacon) was specific for CD11b (Human ITGAM M-008008-01). A nontargeting siRNA control pool (D-001206-13-05; Dharmacon) served as a control. The transfection efficiency was determined by flow cytometry of cells transfected with siGlo RISC-Free Control siRNA (D-001600-01; Dharmacon). Silencing of CD11b expression was verified by real-time PCR and flow cytometry.

### Statistical analysis.

The statistical program GraphPad Prism 5 (GraphPad Software, La Jolla, CA, USA) was used for analysis of all data. Repeated-measures analysis of variance (ANOVA), followed by a Bonferroni posttest or a two-tailed paired *t* test, was used to test for statistical significance. Results were considered statistically significant if the *P* value was <0.05. All experiments were performed a minimum of four times using cells derived from different blood donors or different cell line passages. When experimental values were normalized, the mean of free virus or mock treatment was set to 1. qPCR results were normalized for variation between plates, as previously described (35). In brief, each value was subtracted from the average of all values, and then the values obtained were divided by the average of mock treatment or free virus, depending on the experiments.

## RESULTS

### Complement opsonization of HSV-2 increased infection of immature dendritic cells, whereas the presence of neutralizing HSV-1 or HSV-2 Abs abolished infection.

**FIG 1:**
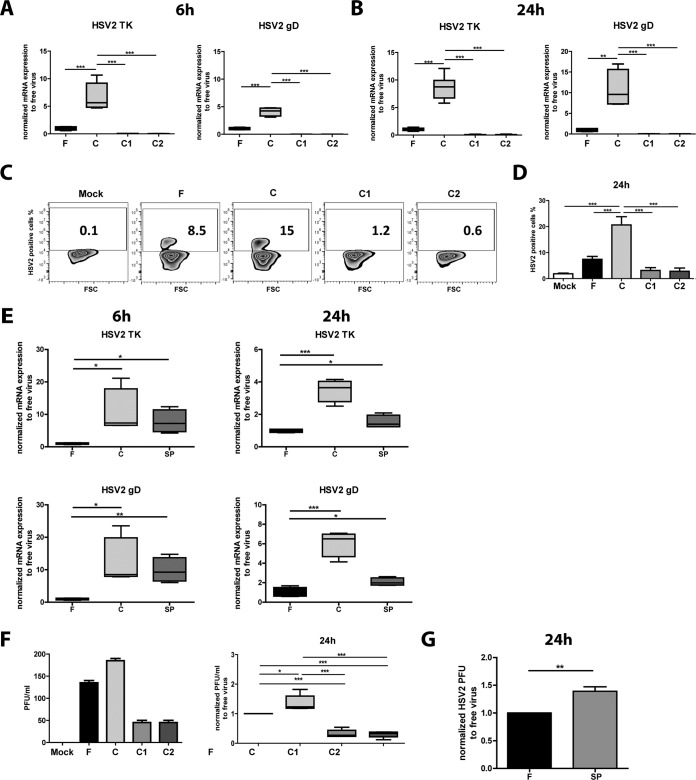
Complement opsonization of HSV-2 increased infection in immature DCs. Immature DCs (10^6^/ml) were exposed to mock treatment or free HSV-2 (F), HSV-2 complement opsonized with HSV-1/2-seronegative serum (C), HSV-2 opsonized with HSV-1 (C1)- or HSV-2 (C2)-seropositive serum, or HSV-2 opsonized with SP from an HSV-1/2-seronegative donor at an MOI of 3 for 6 h or 24 h. (A and B) mRNA expression levels at 6 h and 24 h for HSV-2 TK and gD were assessed by qPCR. The qPCR values were normalized with free-virus mean values set to 1. (C and D) HSV-2 infection was assessed by flow cytometry at 24 h using a PAb against HSV-2, and representative dot plots (C) and percentages of HSV-2-positive cells (D) are shown (means plus standard errors of the mean [SEM]). The data are shown as box-and-whisker plots of the results of 4 to 6 independent experiments. (E) PFU assays were performed to assess productive HSV-2 infection, i.e., production of infectious viral particles by DCs. Supernatants (24 h) from DCs exposed to mock treatment, F, C, C1, and C2 were tested, and the number of HSV-2 PFU per milliliter was calculated. The results of a representative experiment (left) and normalized experiments (*n* = 5) with PFU values of free-virus set to 1 (right) are shown. The data are shown as box-and-whisker plots of the results of 5 independent experiments. (F) mRNA expression levels at 6 h and 24 h for HSV-2 TK and gD were assessed by qPCR. The qPCR values were normalized with free-virus mean values set to 1. (G) PFU assays were performed to assess productive HSV-2 infection in DCs. Supernatants (24 h) from DCs exposed to F and SP were tested, and the number of HSV-2 PFU per milliliter was calculated. Shown are the results of experiments (*n* = 4) normalized with PFU values of free virus set to 1. *, *P* < 0.05; **, *P* < 0.005; ***, *P* < 0.0005.

HSV-2 has the ability to infect immature monocyte-derived DCs (26, 27) and to give rise to a low level of productive infection (26). HSV-2 expresses gC, a glycoprotein that neutralizes complement factor C3 on the viral envelope (7–11). Here, we have assessed how complement opsonization affected the HSV-2 infection of immature monocyte-derived DCs by mock infecting them or infecting them with F-HSV-2, C-HSV-2, C1-HSV-2, or C2-HSV-2. The mRNA expression levels of HSV-2 thymidine kinase (TK), an enzyme involved in viral nucleotide biosynthesis and DNA metabolism, and glycoprotein D (gD), a receptor that is part of the core fusion machinery and important in viral entry, were assessed by qPCR at 6 h and 24 h postinfection (Fig. 1A and B). C-HSV-2 induced significantly higher mRNA expression of HSV-2 TK and gD at both 6 h and 24 h in DCs than free virus (Fig. 1A and B). The presence of HSV-1- or HSV-2-specific antibodies in the serum used for opsonization almost abolished infection, as seen in the decreased mRNA expression levels of TK and gD compared to free virus and C-HSV-2 at both time points (Fig. 1A and B). The gene expression pattern of the HSV-2 protein ICP0 was similar to that of TK and gD (see Fig. S1A in the supplemental material). The mRNA profiles matched the profiles of HSV-2 proteins expressed at 24 h as assessed by flow cytometry, with 2-fold-higher levels of HSV-2^+^ cells in the C-HSV-2-infected group than in the free-HSV-2-infected group (Fig. 1C and D). A similar protein expression pattern was obtained for HSV-2 ICP8 (see Fig. S1B in the supplemental material). To verify the results with a source of complement that exists at the site of infection, we assessed the effect opsonization with SP exerted on HSV-2 infection of DCs. SP-HSV-2 induced greater infection of DCs than free virus at both 6 h and 24 h (Fig. 1E) at the gene transcript level. This indicated that, even if less complement was present in the seminal plasma (36), the complement-opsonized virus still had higher capacity to infect DCs than free virus.

HSV-2 infection is known to induce apoptosis in DCs (5), and we could confirm this effect in our system with higher levels of dead and apoptotic cells for F-HSV-2- and C-HSV-2-infected cells than for C1-HSV-2-, C2-HSV-2-, and mock-infected cells (data not shown; see Fig. S1C in the supplemental material). As an infection control, we assessed the effects of UV-inactivated HSV-2 and found that the inactivation abolished infection (see Fig. S2 in the supplemental material), which is consistent with previous findings (26).

### Complement opsonization of HSV-2 enhanced the productive infection of dendritic cells.

To assess the levels of productive HSV-2 infection in DCs, we determined the number of released infectious virions in the supernatants at 24 h postinfection by plaque-forming assay. After 24 h, immature DCs infected with C-HSV-2 had significantly higher production and release of viral particles than free virus, C1-HSV-2, and C2-HSV-2 (Fig. 1F). A similar pattern was obtained when HSV-2 was opsonized with seminal plasma (Fig. 1G).

### Complement opsonization of HSV-2 increased inflammatory and antiviral transcripts in dendritic cells.

**FIG 2:**
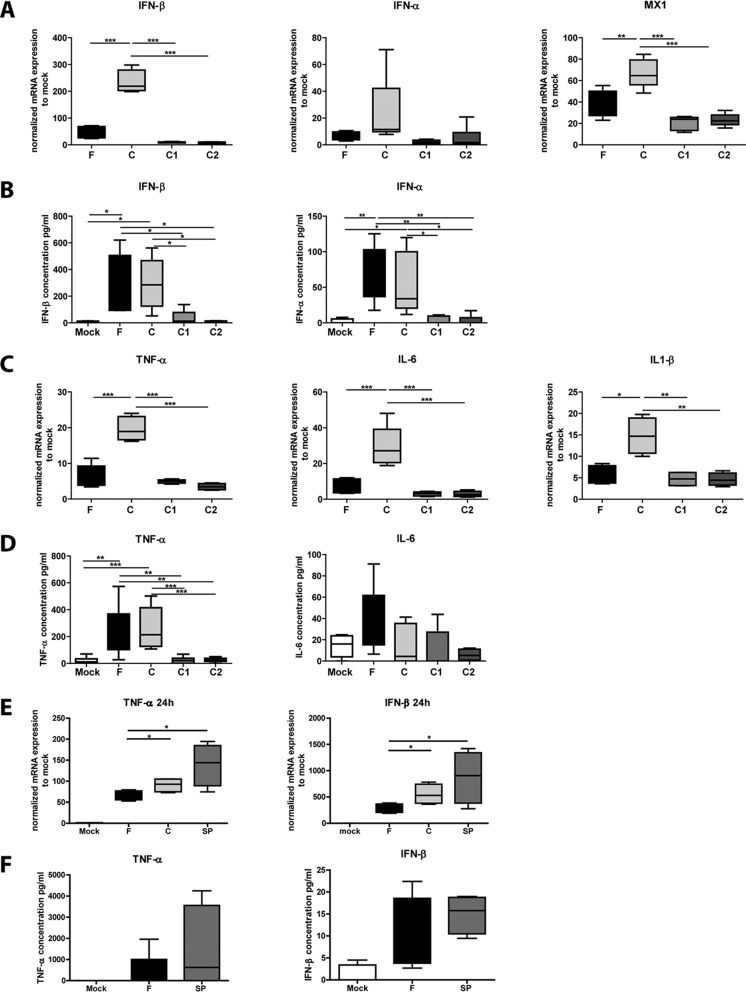
Complement-opsonized HSV-2 induced higher mRNA levels of inflammatory and antiviral factors in immature DCs than free virus. DCs (10^6^/ml) were exposed to free HSV-2 (F), complement-opsonized HSV-2 (C), HSV-2 opsonized with both complement and specific antibodies against HSV-1 (C1) or HSV-2 (C2) at an MOI of 3 or mock treated for 24 h. (A and C) mRNA expression of antiviral factors IFN-β, IFN-α, and MX1 (A) and inflammatory factors TNF-α, IL-6, and IL-1β (C) was determined by qPCR. The qPCR values were normalized with mock treatment mean values set to 1. (B and D) Protein levels of IFN-α and IFN-β (B) and IL-6 and TNF-α (D) were assessed in the supernatants from DCs by ELISA. The data are shown as box-and-whisker plots of the results of 5 or 6 independent experiments. (E) DCs (10^6^/ml) were exposed to free HSV-2 (F), complement-opsonized HSV-2 (C), or HSV-2 opsonized with SP from an HSV-1/2-seronegative donor at an MOI of 3 or mock treated for 24 h. mRNA expression of the inflammatory factor TNF-α and the antiviral factor IFN-β were determined by qPCR. The qPCR values were normalized with mock treatment mean values set to 1. The data are shown as box-and-whisker plots of the results of 5 or 6 independent experiments. *, *P* < 0.05; **, *P* < 0.005; ***, *P* < 0.0005.

Seeing that complement-opsonized HSV-2 enhanced infection, we assessed the effects the opsonized virions had on the ability of DCs to take action against the infection by producing antiviral and inflammatory factors. We have recently demonstrated enhanced infection and suppressed antiviral and inflammatory responses in DCs exposed to complement-opsonized HIV-1 (30). The mRNA expression levels of antiviral factors IFN-β, IFN-α, and MX1 and inflammatory factors TNF-α, IL-6, and IL-1β were assessed for free and opsonized HSV-2. The mRNA levels of IFN-β and MX1 induced by C-HSV-2 were significantly higher than those induced by F-HSV-2 at 24 h (Fig. 2A), but no significant difference in IFN-α levels was observed between F-HSV-2 and C-HSV-2 (Fig. 2A). The presence of HSV-1- or HSV-2-specific antibodies decreased IFN-β expression at 24 h (Fig. 2A). Surprisingly, similar protein levels of the antiviral factors IFN-β and IFN-α were produced by DCs after F-HSV-2 and C-HSV-2 infection (Fig. 2B), whereas the protein levels were significantly lower for C1-HSV-2 and C2-HSV-2 than for F-HSV-2 (Fig. 2B). C-HSV-2 significantly increased the mRNA levels of the inflammatory factors TNF-α, IL-6, and IL-1β in DCs at 24 h compared to F-HSV-2 (Fig. 2C).

In addition, the mRNA expression of the chemokines CCL3 and CXCL8 had a pattern similar to that of the inflammatory factors at 24 h postinfection (see Fig. S3 in the supplemental material). The secretion of IL-6 and TNF-α in DC supernatants were assessed at 24 h postinfection. Complement-opsonized virus and F-HSV-2 induced similar levels of these inflammatory factors (Fig. 2D), The reason for this discrepancy between mRNA and protein expression levels could be due to a more suppressive cellular function in C-HSV-2-infected DCs than in free virus and/or activation of a different regulation of protein transcription by microRNAs in C-HSV-2- than in free-HSV-2-infected DCs. The TNF-α levels induced by C-HSV-2 were much higher than the levels for C1-HSV-2 and C2-HSV-2 (Fig. 2D). Moreover, similar to serum, HSV-2 opsonized with seminal plasma induced higher TNF-α and IFN-β mRNA levels in DCs than free HSV-2 (Fig. 2E). At the protein level, seminal-plasma-opsonized virus tended to increase the secretion of TNF-α and IFN-β compared to free virus, but the data were not statistically significant and exhibited large variation between donors (Fig. 2F).

### Complement opsonization did not alter the number of HSV-2 particles binding to and taken up by dendritic cells.

**FIG 3:**
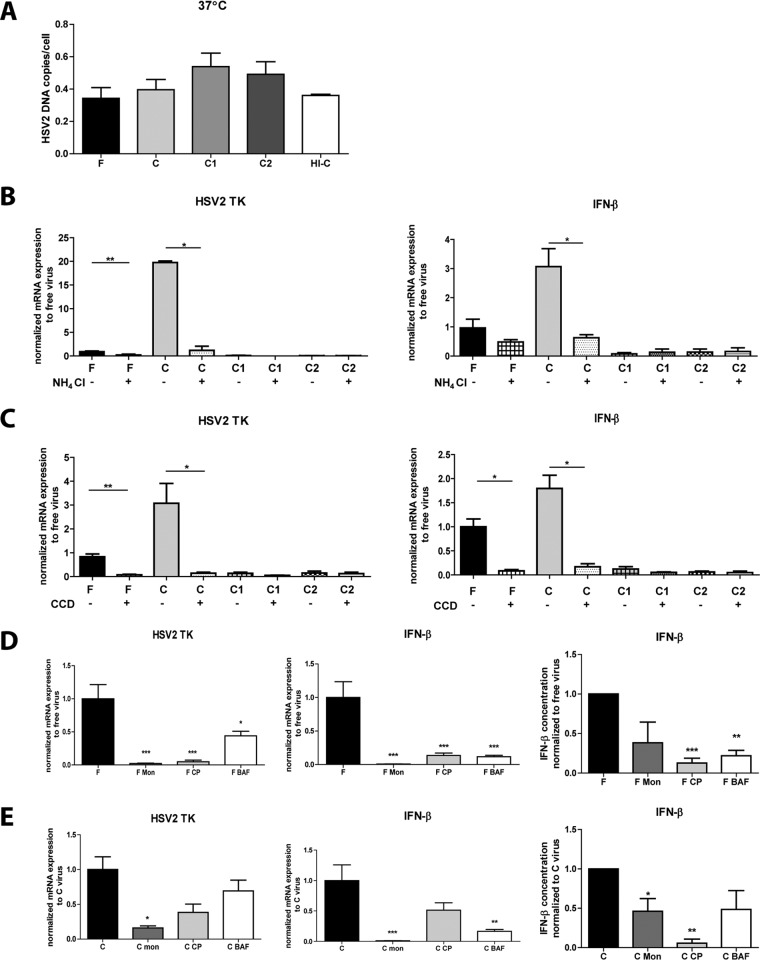
HSV-2 infection of DCs requires endocytosis and endosomal acidification. DCs (10^6^/ml) were exposed to free HSV-2 (F), complement-opsonized HSV-2 (C), HSV-2 opsonized with both complement and specific antibodies against HSV-1 (C1) or HSV-2 (C2), or heat-inactivated C-HSV-2 (HI-C) at an MOI of 3 or mock treated for 2 h or 6 h. (A) Levels of HSV-2 viral DNA copies in immature DCs exposed to F, C, C1, C2, or HI-C for 2 h were assessed by qPCR. (B to E) DCs were preincubated for 30 min with 40 mM NH_4_Cl (B), 10 μM CCD (C), 50 nM BAF, 4 μl/ml Mon, and 6.25 μg/ml CP (D and E) before HSV-2 infection. (B and C) mRNA expression levels of HSV-2 TK and IFN-β were determined by qPCR. The values have been normalized with free-virus mean values set to 1, and the data are shown as means plus SEM of the results of 4 or 5 independent experiments. (D and E) mRNA expression levels of HSV-2 TK and IFN-β were determined by qPCR at 6 h. The values have been normalized with free-virus or complement-opsonized-virus mean values set to 1. The protein levels of IFN-β were assessed in the supernatants from DCs by ELISA at 6 h. The data are shown as means plus SEM of the results of 4 or 5 independent experiments. *, *P* < 0.05; **, *P* < 0.005; ***, *P* < 0.0005.

To examine whether the higher level of infection induced by C-HSV-2 was due to altered binding and internalization mechanisms, the levels of bound and internalized F-HSV-2, C-HSV-2, C1-HSV-2, C2-HSV-2, and heat-inactivated C-HSV-2 (HI-C) in DCs were assessed by determining the number of viral-DNA copies per milliliter after 2 h at 4°C (data not shown) or 37°C (Fig. 3A). The levels of bound and internalized virions were similar for all virus groups, with a tendency toward greater viral uptake for C1-HSV-2 and C2-HSV-2 (Fig. 3A).

### Inhibition of endocytosis and endosomal acidification impeded complement-opsonized HSV-2 infection of dendritic cells.

HSV infection of epithelial cell lines has previously been shown to require, in part, an acid endosomal compartment and endocytosis (37, 38); therefore, we examined the effects that inhibitors of acidification, NH_4_Cl and BAF; inhibitors of endocytosis, CCD and CP; and Mon, a carboxylic ionophore, exerted on the HSV-2 infection of DCs. Drugs were used at concentrations previously shown to block viral uptake and endosomal acidification in DCs or other cell types (39–42) without affecting viral infectivity. Neutralization of endosomal acidification with NH_4_Cl decreased the F-HSV-2 and C-HSV-2 infection of DCs, with almost 20-fold-decreased C-HSV-2 infection at 6 h postinfection (Fig. 3B). Moreover, the mRNA expression level of the antiviral response, as measured by IFN-β, gave the same profile as infection with significantly decreased C-HSV-2 levels (Fig. 3B). The inhibition of DC endocytosis with CCD more or less abolished F-HSV-2 and C-HSV-2 infection at 6 h (Fig. 3C), and the same effects were seen for the antiviral response (Fig. 3C).

F-HSV-2 infectivity and antiviral response were also inhibited by monensin, chlorpromazine, and bafilomycin (Fig. 3D), further confirming the involvement of endosomal acidification and clathrin-mediated endocytosis in the process of HSV-2 infection of DCs. Our finding regarding the effect of bafilomycin on HSV-2 infection correlated with a previous finding (42). C-HSV-2's infectivity was also inhibited by monensin and chlorpromazine, but to a significant level only by monensin, whereas bafilomycin had no effect (Fig. 3E). The inhibitors also decreased the antiviral response induced by C-HSV-2 (Fig. 3E). Treatment with these inhibitors had no direct effect on viral infectivity, i.e., the ability of HSV-2 to infect, or on the DCs' baseline expression of antiviral and inflammatory factors (data not shown).

### The elevated HSV-2 infection in dendritic cells induced by complement-opsonized virions required functional-complement activation.

**FIG 4:**
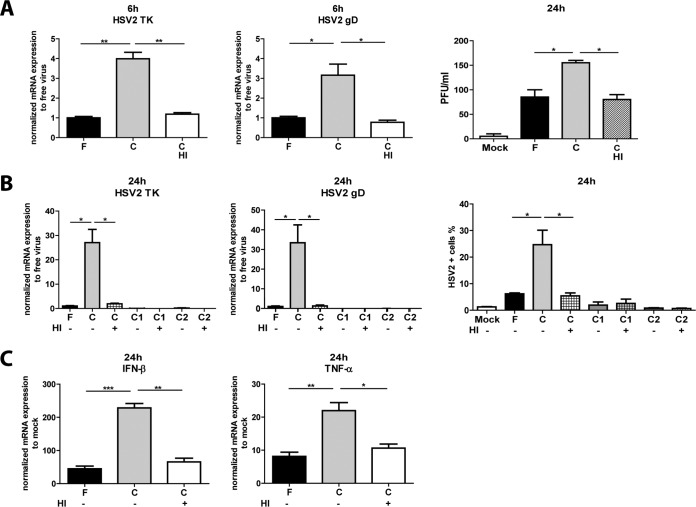
Complement is required for the elevated HSV-2 infection in DCs induced by complement-opsonized virions. DCs (10^6^/ml) were exposed to mock treatment or free HSV-2 (F), HSV-2 complement opsonized with HSV-1/2-seronegative serum (C), HSV-2 opsonized with HSV-1 (C1)- or HSV-2 (C2)-seropositive serum or heat-inactivated serum (HI) at an MOI of 3 for 6 h and 24 h. (A) mRNA expression levels for HSV-2 TK and gD assessed by qPCR at 6 h. PFU assays were performed to test productive infection at 24 h, and the values are expressed as PFU per milliliter (the results of a representative experiment are shown). (B) mRNA expression levels for HSV-2 TK and gD were assessed by qPCR or by HSV-2 protein expression determined by flow cytometry at 24 h using a PAb against HSV-2 to assess the percentage of HSV-2-positive cells. (C) mRNA expression levels for IFN-β and TNF-α were assessed by qPCR at 24 h. The qPCR values were normalized with mock treatment set to 1. The data are shown as means plus SEM of the results of 4 or 5 independent experiments. *, *P* < 0.05; **, *P* < 0.005; ***, *P* < 0.0005.

Activation of the complement cascade is inhibited by heat inactivation of serum, and we confirmed the involvement of complement in the enhanced infection seen for C-HSV-2 by using heat-inactivated serum. Opsonization of HSV-2 with heat-inactivated serum gave the same binding and internalization level as F-HSV-2 and C-HSV-2 (data not shown). Heat inactivation of complement-opsonized virus significantly decreased the mRNA expression of HSV-2 TK and HSV-2 gD compared to C-HSV-2, and the levels were similar to that of free virus at 6 h (Fig. 4A). The same pattern was seen for productive infection (Fig. 4A) and for HSV-2 protein expression at 24 h (Fig. 4B). Inactivation had no significant effects on C1-HSV-2 and C2-HSV-2 infection levels, on TK and gD mRNA, or on HSV-2 protein levels (Fig. 4B). In the cases of antiviral and inflammatory factors, the pattern was similar, and HSV-2 opsonized with heat-inactivated serum restored IFN-β and TNF-α to the levels of free virus at 24 h (Fig. 4C).

### The elevated infection induced by complement-opsonized HSV-2 in dendritic cells required functional complement receptor 3.

**FIG 5:**
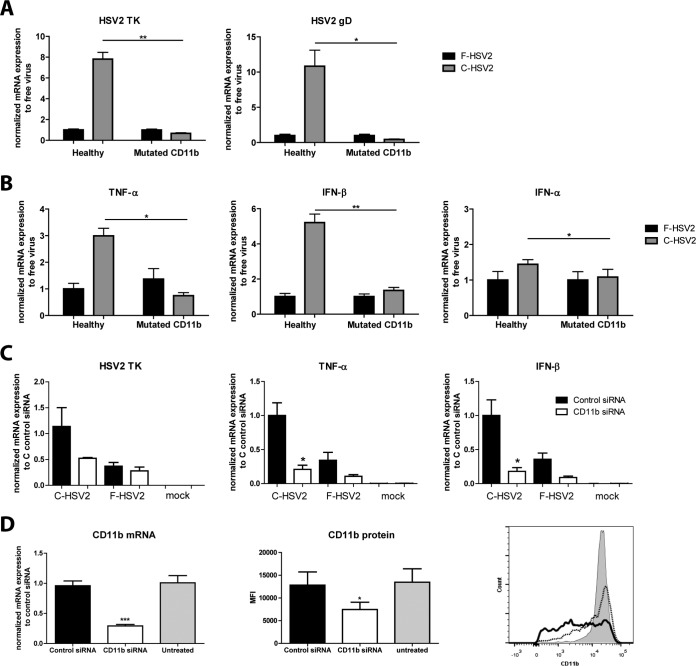
Functional complement receptor 3 was required for the elevated HSV-2 infection in DCs induced by complement-opsonized virions. (A and B) DCs (10^6^/ml) propagated from individuals with mutated nonfunctional CD11b, i.e., CR3, were exposed to mock treatment or to free HSV-2 (F) or HSV-2 complement opsonized with HSV-1/2-seronegative serum (C) at an MOI of 3 for 24 h. mRNA expression levels for HSV-2 TK, gD, TNF-α, IFN-β, and IFN-α were assessed by qPCR at 24 h. The qPCR values were normalized with free-virus mean values set to 1. The data are shown as means plus SEM of the results of 3 to 5 independent experiments. (C and D) DCs (10^5^/100 μl) were transfected with CD11b siRNA or control siRNA at day 2 or 3 and used for experiments 2 days after transfection. (C) mRNA expression levels for HSV-2 TK, TNF-α, and IFN-β were assessed by qPCR at 24 h. The qPCR values were normalized with the C-HSV-2 control siRNA mean value set to 1. (D) CD11b expression was evaluated by qPCR (left) and flow cytometry (right). A flow cytometry representative histogram (gray shading, untreated; dotted line, control siRNA; solid line, CD11b siRNA) and a summary graph are shown. The data are shown as means plus SEM of the results of 3 to 5 independent donor experiments. *, *P* < 0.05; **, *P* < 0.005; ***, *P* < 0.0005.

CR3 is exploited by several pathogens for suppression of innate responses (43, 44). DC expression of CR3 is required for complement-opsonized HIV's augmentation of infection (30); consequently, we examined whether the enhanced HSV-2 infection of DCs by complement-opsonized HSV-2 also was dependent on viral binding to CR3. Here, we used DCs derived from individuals with SLE with mutated CR3 alpha-integrin CD11b (rs1143697; R77H), which gives decreased expression of CD11b and impairs signaling through CR3 (30, 45, 46). Free HSV-2 produced the same level of infection in DCs derived from individuals with SLE as in cells from healthy donors (see Fig. S4 in the supplemental material). The enhanced infection seen for C-HSV-2 in DCs from healthy individuals was abolished when the DCs had dysfunctional CR3 (Fig. 5A). Moreover, the enhanced gene expression of inflammatory and antiviral factors normally seen for C-HSV-2-exposed DCs was not detected when DCs with mutated CR3 were used (Fig. 5B). To confirm the findings from the SLE patient-derived DCs with dysfunctional CR3, we knocked down CD11b expression with siRNA and assessed infection, inflammation, and antiviral responses (Fig. 5C and D). The knockdown of CD11b abolished increased C-HSV-2 infection, whereas this increase was still present in the control siRNA-transfected cells (Fig. 5C). Moreover, the higher gene expression of inflammatory and antiviral factors seen for C-HSV-2-exposed DCs was not present in the CD11b knockdown DCs (Fig. 5C).

## DISCUSSION

Langerhans cells (LCs) and DCs are among the initial responder cells during the establishment of HSV-2 infection in the mucosa and are involved in the induction of HSV-2-specific adaptive immunity via DC cross-presentation (5, 47). At the site of infection, the HSV-2 virions are exposed to soluble factors, such as complement and antibodies, which should influence the effects virions exert on their target cells, as well as local infection. We found that HSV-2's capacity to utilize the complement system, using serum or seminal plasma, enhanced the virus' ability to directly infect DCs and that the enhanced infection required functional CR3. The presence of HSV-1- or HSV-2-specific antibodies during opsonization more or less abolished the ability of HSV-2 to infect DCs. The HSV-2 infection of DCs required endocytosis of virus particles and acidification of the endosomal compartment. Complement-opsonized HSV-2 induced higher mRNA expression but not statistically significantly higher protein secretion of antiviral and inflammatory factors in the DCs than free HSV-2.

HSV-2 has the ability to infect several cell types, such as epithelial cells, nerve cells, and DCs, located in the genital mucosa (1, 5), and *in vitro* studies have shown that immature monocyte-derived DCs and LCs are highly susceptible to HSV-2 infection (5, 26, 27, 48–50). Our findings support the notion that HSV-2 infects DCs and that this gives rise to a low level of productive infection. The low level of productive infection is not surprising, since previous studies have found HSV-2 and HSV-1 infection of DCs to be predominantly abortive (26, 51–53). This should be the explanation for the discrepancies we see between the high levels of viral-mRNA transcripts and the low levels of infectious HSV-2 produced by infected DCs. One additional explanation could be the existence of cellular inhibitors of infection that influence events after mRNA transcription, i.e., inhibiting the production of viral proteins and/or infective virions.

The first steps in HSV infection of target cells, i.e., binding and uptake of viral particles, have been investigated, and almost all studies focused on HSV-1 and epithelial cell lines (37–39, 54). The mechanism for HSV-1 and HSV-2 uptake in epithelial cells is rapid endocytosis (39). HSV-1 endocytosis requires several of the viral envelope glycoproteins, i.e., gB, gD, and gH-gL (55, 56), and in keratinocytes, HSV-1 uptake is a cholesterol- and dynamin-mediated process. In the endosomal compartment, HSV-1 and HSV-2 utilize the viral gD receptor to enter the host cell cytosol (39) and, in the case of HSV-1 dynamin, to infect the cell (57). The binding and uptake of HSV-2 by DCs has not been examined previously, and we found that complement opsonization had no effect on the number of HSV-2 virions bound or internalized. HSV-2 infection, for both free and complement-opsonized virions, was dependent on endocytosis and endosomal acidification. Interestingly, infection with free virus involved clathrin-dependent endocytosis, whereas complement-opsonized virus seemed to utilize a clathrin-independent mechanism for infection. In addition to suppressing infection, the neutralization of endosomal acidification decreased the antiviral responses in DCs. This indicates that the antiviral responses require degradation of the viral particles and/or active infection of the DCs. TLR3 and TLR2 have been suggested to be important pattern recognition receptors (PRRs) in the immunological control of HSV-2 infection (58–61). Furthermore, sensors such as STING and DAI can also sense HSV-2 (62). The exact PRRs involved in the activation of the antiviral and inflammatory responses in the human DCs in our system of HSV-2 infection remain to be determined. Our finding that HSV-2 antiviral responses in DCs required endocytosis of the virus into an acidified endosomal compartment suggests the involvement of endosomal and/or cytosolic PRRs.

A number of previous studies have suggested that prior oral HSV-1 infection can provide partial protection against genital HSV-2 acquisition (22–24), whereas others have proposed that it has no protective effect (63–65). At present, most evidence indicates that preexisting HSV-1 antibodies do not inhibit infection; rather, they make the HSV-2 infection less pathogenic, with milder symptoms (22–25). We found that the presence of HSV-1-specific antibodies more or less abolished the ability of HSV-2 to infect DCs, and surprisingly, they neutralized the infection with the same efficiency as HSV-2-specific antibodies. The presence of HSV-1 or HSV-2 antibodies did not decrease the uptake of virus by DCs, but rather, slightly increased it, so the absence of infection was not due to inability of the virus to bind and be internalized but rather to neutralization of HSV-2 infectivity. This raises questions such as how the adaptive immune response induced by a prior oral HSV-1 infection affects genital mucosal responses during primary genital HSV-2 infection, i.e., whether the existing HSV-1 antibodies neutralize the virus and render the virions less cytopathic and suppressive of DC functionality, and this needs further elucidation.

HSV-2 uses many strategies to establish persistent infection and inhibition of complement activation/cascade, and antibody binding by gC and gE/gI proteins on the viral surface are part of these mechanisms (7, 66). gC on HSV-2 (gC2) and HSV-1 (gC1) both bind C3b (8–10), and HSV-1 gC also interferes with the binding of C5 and properdin to C3b (12, 15, 67, 68). In fact, HSV-1 gC1 contains a C5- and P-interacting domain that accelerates the decay of the alternative complement pathway C3 convertase, and this domain is important in modulating complement activity, since HSV-1 lacking the domain is more readily neutralized by complement alone and is significantly less virulent than wild-type virus *in vivo*. Interestingly, this domain is absent in HSV-2 gC2, suggesting that the mechanism by which HSV-2 evades the complement cascade may be distinct from that of HSV-1 (12, 14, 69).

In our study, we found that complement fragments binding to HSV-2 enhanced the direct and productive infection of DCs. The enhanced HSV-2 infection of DCs induced when the virus is complement opsonized has also been observed for HIV-1 by our group and by others (30, 70, 71). In the case of HIV-1 and the bacterium Francisella tularensis, complement opsonization suppresses the pathogen-induced antiviral and inflammatory immune response by CR3-mediated modulation of Toll-like receptor (TLR) signaling pathways (30, 43). Even if the enhanced infection achieved by complement-opsonized HSV-2 was due to CR3 interaction, it did not suppress the secretion of antiviral and inflammatory factors. The mechanism behind these differences between pathogens could be diversity of receptors, such as the type of pattern recognition receptors activated, due to the presence of different pathogen-associated molecular patterns (PAMPs), in combination with CR3, giving rise to distinct signaling and activation of DCs.

The HSV-2 infection of DCs in our hands induced an array of inflammatory and antiviral factors with higher mRNA levels induced by the complement-opsonized viruses than by free virus, whereas the protein expression levels were the same. These discrepancies between mRNA and protein expression levels could be due to a more suppressive cellular function in C-HSV-2-infected DCs than in free-virus-infected DCs and/or activation of a different regulation of protein transcription by microRNAs in C-HSV-2- versus free-HSV-2-infected DCs.

In conclusion, HSV-2 exploitation of innate defense, i.e., the complement system, enhanced the virus' ability to infect DCs. The presence of HSV antibodies clearly renders the virus virtually noninfectious and less toxic to DCs and should function as a source of antigens for activation of adaptive immune responses. We clearly demonstrate the importance of studying HSV-2 infection under conditions that reflect the *in vivo* situation, i.e., virions covered in complement fragments or complement fragments and antibodies, as these factors have a profound effect on the virus' interaction with the host.

## Supplementary Material

Supplemental material
