# Supplementary material for: Complement Opsonization Promotes Herpes Simplex Virus 2 Infection of Human Dendritic Cells
Source: J Virol. 2016 Apr 29;90(10):4939–50. doi: 10.1128/JVI.00224-16 (PMC4859714; doi:10.1128/JVI.00224-16)
Supplement: Supplemental material [file supp_90_10_4939__index.html]

Complement Opsonization Promotes Herpes Simplex Virus 2 Infection of Human Dendritic Cells — Supplemental material 

# Complement Opsonization Promotes Herpes Simplex Virus 2 Infection of Human Dendritic Cells

## Supplemental material

- Supplemental file 1 -

  Table S1 (Primer sequences.)

  Fig. S1 (DCs were mock infected or exposed to free HSV-2, HSV-2 complement opsonized with HSV-1/2-seronegative serum, HSV-2 opsonized with HSV-1, or HSV-2-seropositive serum for 24 h.)

  Fig. S2 (DCs were mock infected or exposed to free UV-inactivated HSV-2, and mRNA expression levels for HSV-2 TK were assessed by qPCR or flow cytometry.)

  Fig. S3 (DCs were mock infected or exposed to free HSV-2 and HSV-2 complement opsonized with HSV-1/2-seronegative serum for 24 h.)

  Fig. S4 (DCs from healthy donors and donors with SLE were exposed to free HSV-2 for 24 h.)

  PDF, 371K
