# Supplementary material for: Complement Opsonization Promotes Herpes Simplex Virus 2 Infection of Human Dendritic Cells
Source: J Virol. 2016 Apr 29;90(10):4939–50. doi: 10.1128/JVI.00224-16 (PMC4859714; doi:10.1128/JVI.00224-16)
Supplement: Supplemental material [file JVI.00224-16_zjv999091621so1.pdf]

Supplementary Table 1. Primer sequences.

**GAPDH**	**F** CCA CCA TGG AGA AGG CTG GGG CTC<br>**R** AGT GAT GGC ATG GAC TGT GGT CAT
**β-actin**	**F** AGA GGG AAA TCG TGC GTG AC<br>**R** CAA TAG TGA TGA CCT GGC CGT
**TNF-α**	**F** TCT GGC CCA GGC AGT CAG ATC A<br>**R** CAC GCC ATT GGC CAG GAG GG
**IL-6**	**F** TGG CTG AAA AAG ATG GAT GCT T<br>**R** CAA ACT CCA AAA GAC CAG TGA TGA
**IL-1 β**	**F** CTC CAC CTC CAG GGA CAG GAT ATG<br>**R** CGC TTT TCC ATC TTC TC TTT GGG TAA
**IFN-β**	**F** AGT GTC AGA AGC TCC TGT GGC AA<br>**R** TGG TCA ATG CGG CGT CCT CC
**IFN-α4**	**F** ACC TAG AGG CCG AAG TTC AAG GT<br>**R** GCA CCA GCA CGG CCA TCA G
**MXA**	**F** TGC CAG CCA TCG CCG TCA TC<br>**R** CTG GTC ACG ATC CCG CTG CC
**CCL3**	**F** CGG TGT CAT CTT CCT AAC CA<br>**R** GAC ATA TTT CTG GAC CCA CTC
**CXCL8**	**F** GCC ACCC GAG GAG CAG AGA GGT T<br>**R** ATG AGC AGC AGG CAC AGA AGC G
**CD11b**	**F** GTG AAG CCA ATA ACG CAG C<br>**R** TCT CCA TCC GTG ATG ACA AC
**HSV2 TK**	**F** CGA GCC GAT GAC TTA CTG GC<br>**R** GCT GCG TGT TGT AGA TGT TCG
**HSV2 gD**	**F** AAG CGT GTT TAC CAC ATT CAG CCG<br>**R** TGT GTG ATC TCC GTC CAG TCG TTT
**HSV2 ICP0**	**F** GGT CAC GCC CAC TAT CAG GTA<br>**R** CCT GCA CCC CTT CTG CAT

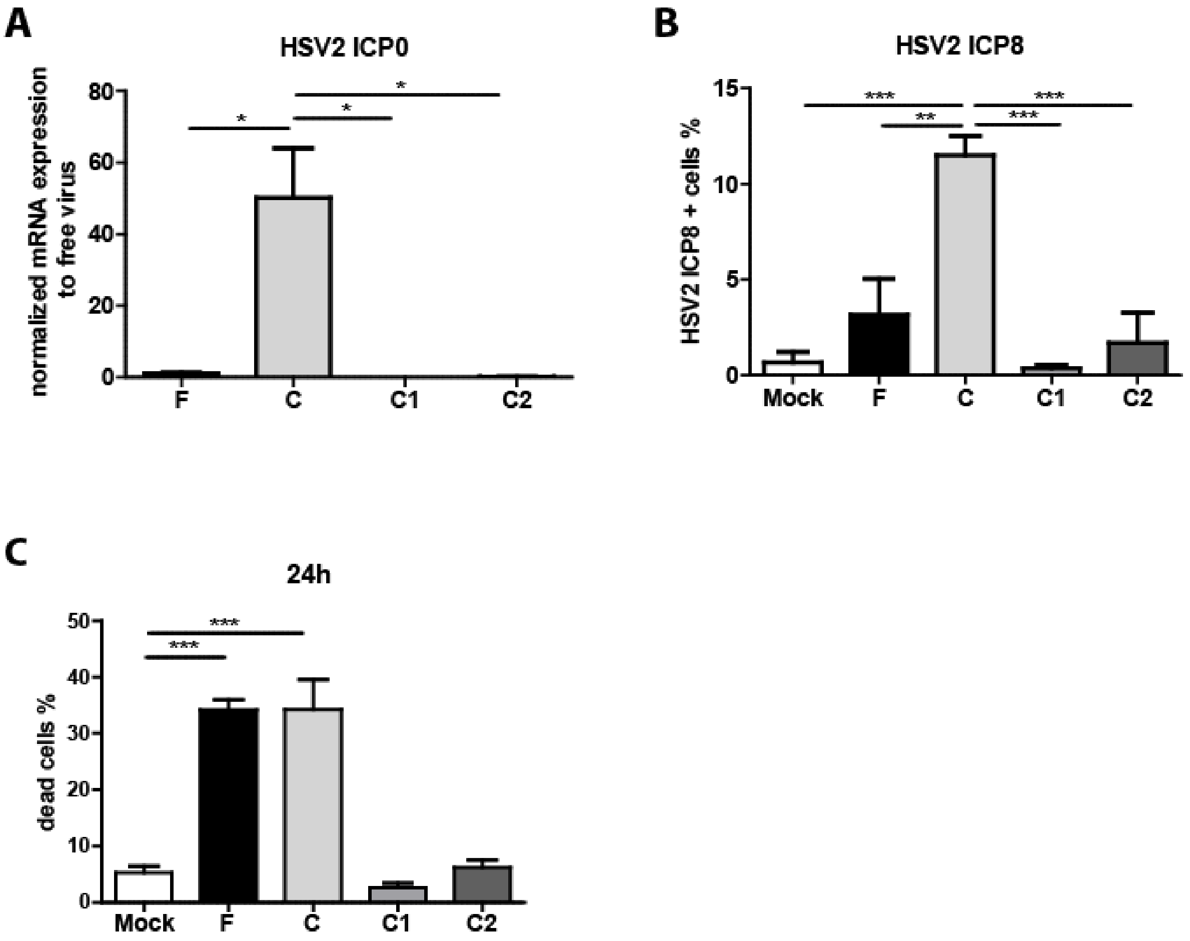

Supplementary figure 1. DCs (10^6/ml) were exposed to mock, 3 MOI free HSV2 (F), HSV2 complement opsonized with HSV1/2 seronegative serum (C), HSV2 opsonized with HSV1 (C1), or HSV2 (C2) seropositive serum for 24h. **(A)** mRNA expression levels of HSV2 ICP0 were assessed by qPCR. qPCR values were normalized with free virus values set to 1. Data are shown as mean + SEM of 4-6 independent experiments. **(B)** Flow cytometry using a mAb against HSV2 ICP8 assessing percentage HSV2 positive cells at 24h. Data are shown as mean + SEM of 4-6 independent experiments. **(C)** Percentage of dead cells at 24h assessed using Zombie Aqua™ Fixable Viability Kit (BioLegend, Europe). Data are shown as mean + SEM of 5 independent experiments. * $p<0.05$. ** $p<0.005$. *** $p<0.0005$.

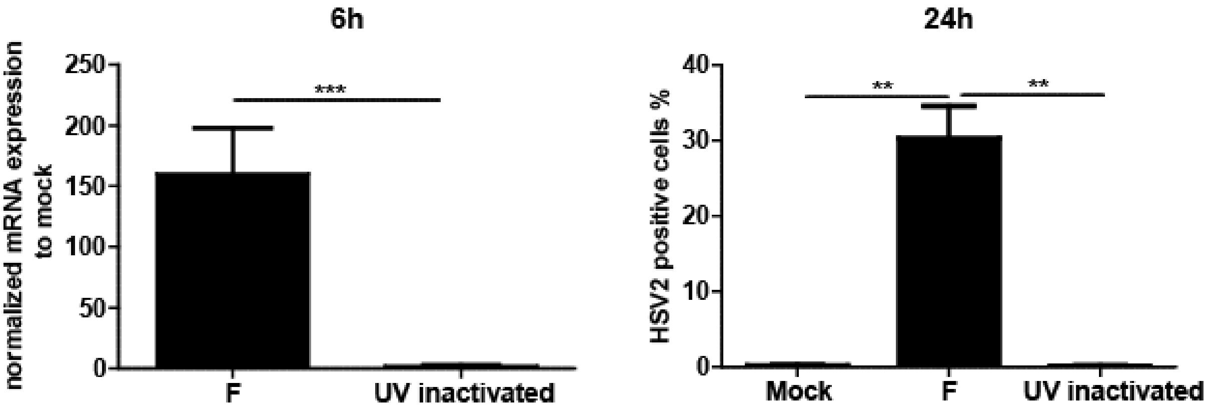

Supplementary figure 2. DCs were exposed to mock, free HSV2 (F), or UV inactivated HSV2 and mRNA expression levels for HSV2 TK were assessed by qPCR at 6h or by flow cytometry at 24h. qPCR values were normalized with mock values set to 1. Data are shown as mean + SEM of 4-6 independent experiments. **$p<0.005$. ***$p<0.0005$.

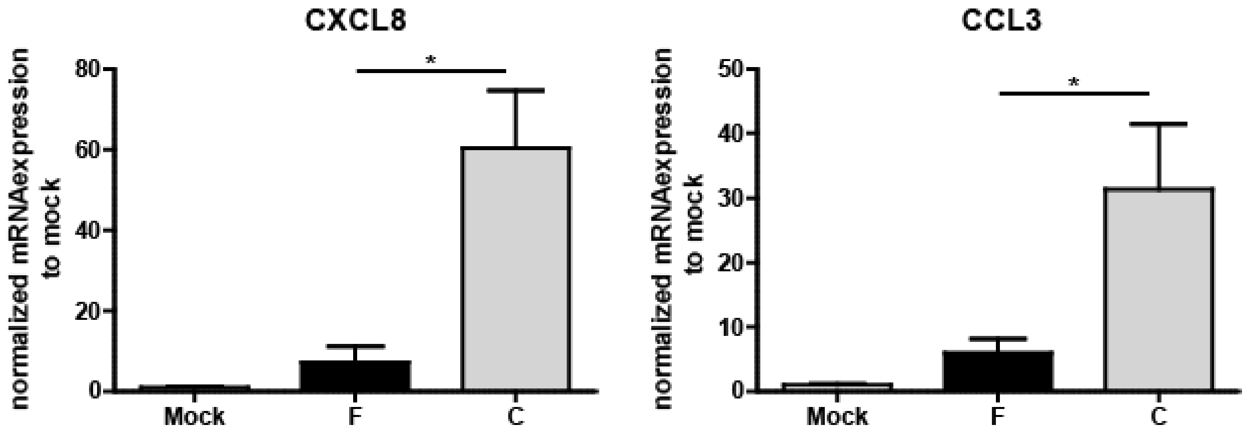

Supplementary figure 3. DCs (10^6/ml) were exposed to mock, 3 MOI free HSV2 (F) and HSV2 complement opsonized with HSV1/2 seronegative serum (C) for 24h. mRNA expression levels of CXCL8 and CCl3 were assessed by qPCR. qPCR values were normalized with mock values set to 1. Data are shown as mean + SEM of 4-6 independent experiments* $p<0.05$.

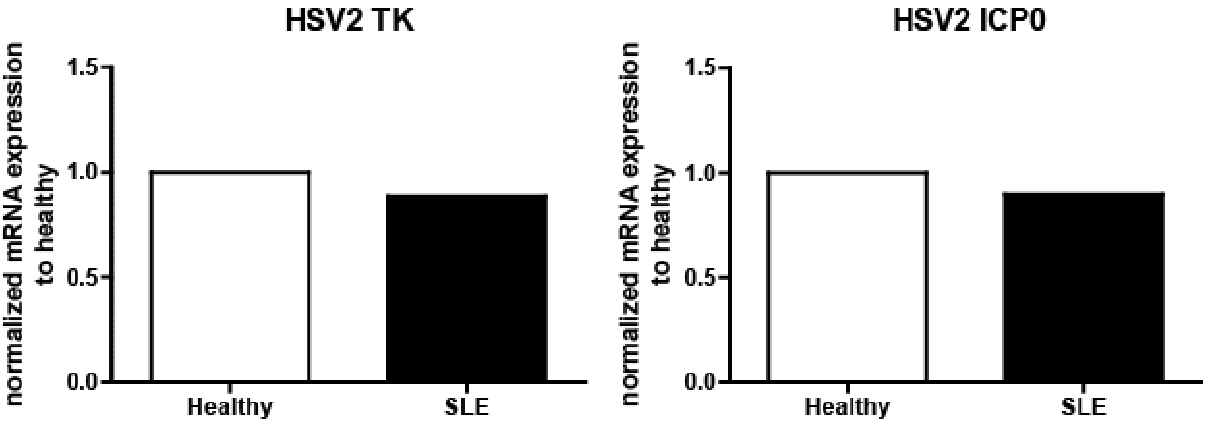

Supplementary figure 4. DCs (10^6/ml) from healthy and SLE donors were exposed to 3 MOI free HSV2 for 24h. mRNA expression levels of HSV2 TK and ICP0 were assessed by qPCR. qPCR values were normalized to the mean of the free virus values from healthy donors set to 1. Data are shown as mean of 4 independent experiments.
